# Supplementary material for: Chromosome-specific differences in the recombination landscape of spontaneous meiotic nondisjunction
Source: Genetics. 2026 Mar 24;233(1):iyag076. doi: 10.1093/genetics/iyag076 (PMC13147521; doi:10.1093/genetics/iyag076)
Supplement: iyag076_Supplementary_Data [file iyag076_supplementary_data.pdf]

Supplementary Information

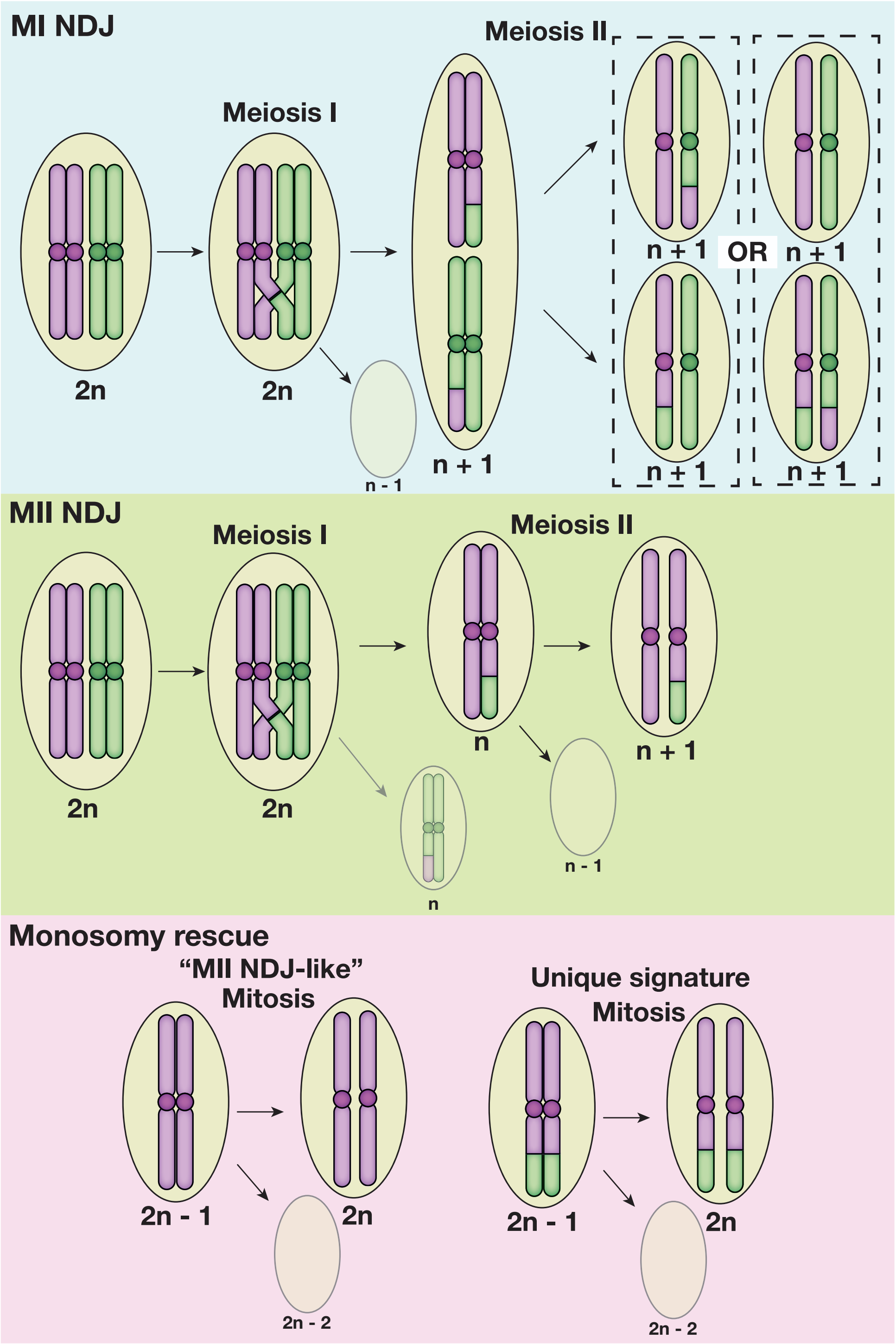

Figure S1. Genotypes resulting from different types of NDJ.
For each scenario, a single crossover is considered. Only chromosome *2* is shown (other chromosomes, not depicted, have segregated normally), with ploidy indicated below each cell. MI NDJ results in heterozygosity across the centromere. A single crossover may be detectable (left set of gametes) or undetectable (right set of gametes) by changes in allele frequency depending on how homologs align on the metaphase plate in meiosis II (only the configuration giving rise to the left set of progeny is shown). MII NDJ results in homozygosity across the centromere, and crossovers are always detectable by allele frequency in meiotic products. Monosomy rescue via mitotic NDJ may either result in progeny with genotypes that are indistinguishable from MII NDJ or progeny with a unique genotype exclusive to mitotic NDJ depending on whether these progeny inherited a recombinant chromosome. Division outcomes that are not represented in the collected progeny are shown with low opacity, and for simplicity the final gametes from these divisions are not shown.

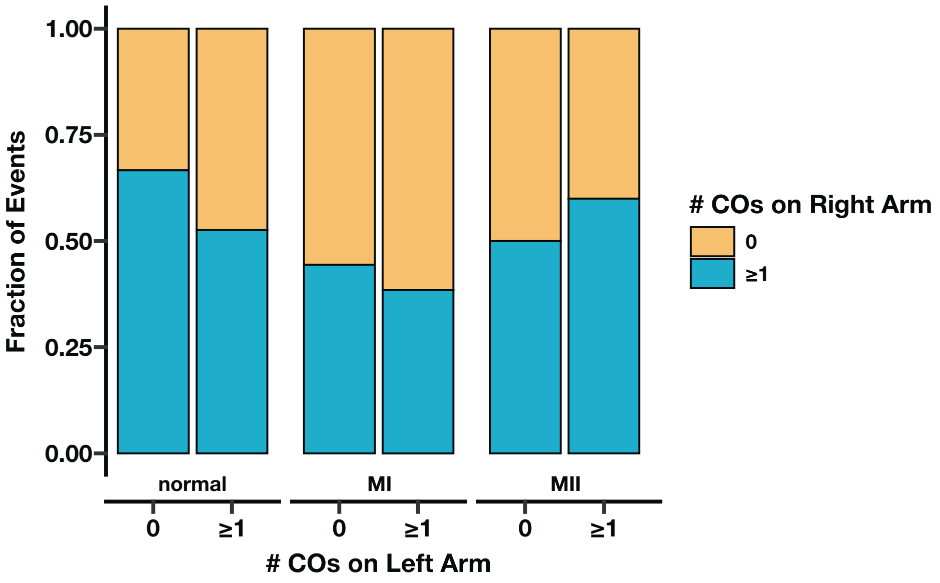

Figure S2. The influence of presence of crossovers on the left arm of chromosome *2* on the presence of crossovers on the right arm appears minimally affected by meiotic NDJ.
The fraction of normal, MI, and MII meiotic NDJ events with zero or at least one detectable crossover on the left arm of chromosome *2* with zero (orange) or at least one (blue) detectable crossover on the right arm of chromosome *2*. Normal meioses were taken from Miller et al. (2016).

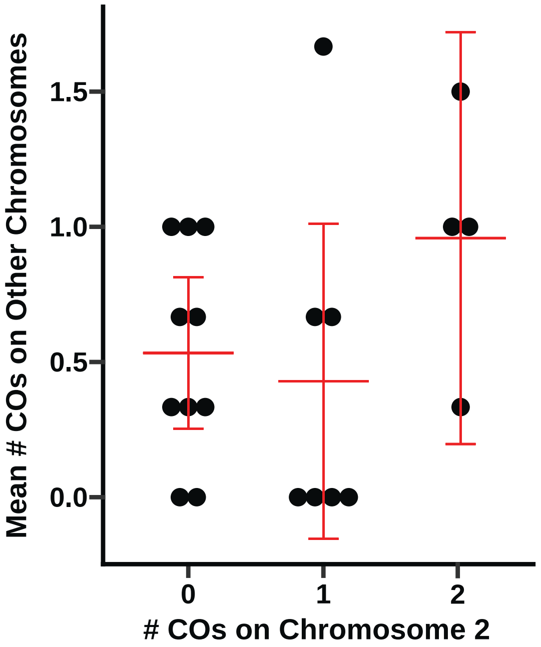

Figure S3. Number of crossovers on chromosome *2* is minimally correlated with recombination rate on other chromosomes in MI NDJ. The mean number of crossovers on chromosomes *X* and *3* relative to how many crossovers were detected on chromosome *2* in each MI NDJ event. Large red bars indicate means, with smaller bars indicating 95% confidence intervals.

Supplementary Tables

Table S1. *Drosophila* stocks.
A table of all stocks used. All stocks are from Bloomington Stock Center, and stock numbers for each genotype are provided.

Table S2. NDJ progeny used for crossover analysis.
A table of all NDJ progeny with their sequencing run information and barcodes used for sample pooling. Barcode numbers and sequences are consistent with those from Oxford Nanopore Technologies Native Barcoding Kit SQL-NBD114.24.

Table S3. Crossovers identified in meiotic NDJ progeny.
All crossovers identified in NDJ progeny are provided with the NDJ male of origin, type of crossover (single or double), and positions of 5' and 3' SNPs used to locate them. In addition, chromosome arms without crossovers are listed with the 5' and 3' SNPs available for that chromosome arm.

Table S1: Drosophila stocks.

Stock	Bloomington Stock Center ID
w ^{1118}	3605
Oregon R	5
C(2)EN, Adc $^{b-1}$ *pr* ^{1}	1112

Table S2: NDJ progeny used for crossover analysis.
All NDJ progeny with their sequencing run information and barcodes used for sample pooling. Barcode numbers and sequences are consistent with those from Oxford Nanopore Technologies Native Barcoding Kit SQL-NBD114.24.

Fly	Library	Instrument Model	Barcode
**NDJ_01**	20240525_2109_X1_FAX64326_9e196595	GridION	1
**NDJ_02**	20240525_2109_X1_FAX64326_9e196595	GridION	2
**NDJ_03**	20240525_2109_X1_FAX64326_9e196595	GridION	3
**NDJ_04**	20240525_2109_X1_FAX64326_9e196595	GridION	4
**NDJ_05**	20240525_2109_X1_FAX64326_9e196595	GridION	5
**NDJ_06**	20240525_2109_X2_FAY63592_dadeda16	GridION	6
**NDJ_07**	20240525_2109_X2_FAY63592_dadeda16	GridION	7
**NDJ_08**	20240525_2109_X2_FAY63592_dadeda16	GridION	8
**NDJ_09**	20240525_2109_X2_FAY63592_dadeda16	GridION	9
**NDJ_10**	20240525_2109_X2_FAY63592_dadeda16	GridION	10
**NDJ_11**	20240525_2109_X3_FAY64702_484c3b86	GridION	11
**NDJ_12**	20240525_2109_X3_FAY64702_484c3b86	GridION	12
**NDJ_13**	20240525_2109_X3_FAY64702_484c3b86	GridION	13
**NDJ_14**	20240525_2109_X3_FAY64702_484c3b86	GridION	14
**NDJ_15**	20240525_2109_X3_FAY64702_484c3b86	GridION	15
**NDJ_16**	20240619_2030_X1_FAX66580_7a420383	GridION	1
**NDJ_17**	20240619_2030_X1_FAX66580_7a420383	GridION	2
**NDJ_18**	20240619_2030_X1_FAX66580_7a420383	GridION	3
**NDJ_19**	20240619_2030_X1_FAX66580_7a420383	GridION	4
**NDJ_20**	20240619_2030_X1_FAX66580_7a420383	GridION	5
**NDJ_21**	20240619_2030_X1_FAX66580_7a420383	GridION	6
**NDJ_22**	20240619_2030_X1_FAX66580_7a420383	GridION	7
**NDJ_23**	20240619_2030_X1_FAX66580_7a420383	GridION	8
**NDJ_24**	20240619_2030_X1_FAX66580_7a420383	GridION	9
**NDJ_25**	20240619_2030_X1_FAX66580_7a420383	GridION	10
**NDJ_26**	20240619_2030_X1_FAX66580_7a420383	GridION	11
**NDJ_27**	20240619_2030_X1_FAX66580_7a420383	GridION	12
**NDJ_28**	20240619_2030_X1_FAX66580_7a420383	GridION	13
**NDJ_29**	20240619_2030_X1_FAX66580_7a420383	GridION	14
**NDJ_30**	20240619_2030_X1_FAX66580_7a420383	GridION	15
**NDJ_31**	20240619_2030_X1_FAX66580_7a420383	GridION	16
**NDJ_32**	20240619_2030_X1_FAX66580_7a420383	GridION	17
**NDJ_33**	20240619_2030_X1_FAX66580_7a420383	GridION	18
**NDJ_34**	20240619_2030_X1_FAX66580_7a420383	GridION	19
**NDJ_35**	20240619_2030_X1_FAX66580_7a420383	GridION	20
**NDJ_36**	20240619_2030_X1_FAX66580_7a420383	GridION	21
**NDJ_37**	20240619_2030_X1_FAX66580_7a420383	GridION	22
**NDJ_38**	20240628_1916_X1_FAX64491_e77a9191	GridION	1

**NDJ_39**	20240628_1916_X1_FAX64491_e77a9191	GridION	2
**NDJ_40**	20240628_1916_X1_FAX64491_e77a9191	GridION	3
**NDJ_41**	20240628_1916_X1_FAX64491_e77a9191	GridION	4
**NDJ_42**	20240628_1916_X1_FAX64491_e77a9191	GridION	5
**NDJ_43**	20240628_1916_X1_FAX64491_e77a9191	GridION	6
**NDJ_44**	20240628_1916_X1_FAX64491_e77a9191	GridION	7
**NDJ_45**	20240628_1916_X1_FAX64491_e77a9191	GridION	8
**NDJ_46**	20240628_1916_X1_FAX64491_e77a9191	GridION	9
**NDJ_47**	20240628_1916_X1_FAX64491_e77a9191	GridION	10
**NDJ_48**	20240628_1916_X1_FAX64491_e77a9191	GridION	11
**NDJ_49**	20240628_1916_X1_FAX64491_e77a9191	GridION	12
**NDJ_50**	20240628_1916_X1_FAX64491_e77a9191	GridION	13
**NDJ_51**	20240628_1916_X1_FAX64491_e77a9191	GridION	14
**NDJ_52**	20240730_1955_X2_FAZ21430_c898a84c	GridION	13
**NDJ_53**	20240730_1955_X2_FAZ21430_c898a84c	GridION	14
**NDJ_54**	20240730_1955_X2_FAZ21430_c898a84c	GridION	15
**NDJ_55**	20240730_1955_X2_FAZ21430_c898a84c	GridION	16
**NDJ_56**	20240730_1955_X2_FAZ21430_c898a84c	GridION	17
**NDJ_57**	20240730_1955_X2_FAZ21430_c898a84c	GridION	18
**NDJ_58**	20240730_1955_X2_FAZ21430_c898a84c	GridION	19
**NDJ_59**	20240730_1955_X2_FAZ21430_c898a84c	GridION	20
**NDJ_60**	20240730_1955_X2_FAZ21430_c898a84c	GridION	21
**NDJ_61**	20240730_1955_X2_FAZ21430_c898a84c	GridION	22
**NDJ_62**	20240730_1955_X2_FAZ21430_c898a84c	GridION	23
**NDJ_63**	20240730_1955_X2_FAZ21430_c898a84c	GridION	24
**NDJ_64**	20241220_1632_MN37708_FAZ20978_f6b22d69	MinION	11
**NDJ_65**	20241220_1632_MN37708_FAZ20978_f6b22d69	MinION	12
**NDJ_66**	20241220_1632_MN37708_FAZ20978_f6b22d69	MinION	13
**NDJ_67**	20241220_1632_MN37708_FAZ20978_f6b22d69	MinION	14
**NDJ_68**	20241220_1632_MN37708_FAZ20978_f6b22d69	MinION	15
**NDJ_69**	20241220_1632_MN37708_FAZ20978_f6b22d69	MinION	16
**NDJ_70**	20241220_1632_MN37708_FAZ20978_f6b22d69	MinION	17
**NDJ_71**	20241220_1632_MN37708_FAZ20978_f6b22d69	MinION	18
**NDJ_72**	20241220_1632_MN37708_FAZ20978_f6b22d69	MinION	19
**NDJ_73**	20241220_1632_MN37708_FAZ20978_f6b22d69	MinION	20
**NDJ_74**	20241220_1632_MN37708_FAZ20978_f6b22d69	MinION	21
**NDJ_75**	20241220_1632_MN37708_FAZ20978_f6b22d69	MinION	22
**NDJ_76**	20241220_1632_MN37708_FAZ20978_f6b22d69	MinION	23
**NDJ_77**	20241220_1632_MN37708_FAZ20978_f6b22d69	MinION	24

Table S3: Crossovers identified in meiotic NDJ progeny. All crossovers identified in NDJ progeny are provided with the NDJ male of origin, type of crossover (single or double), and positions of 5' and 3' SNPs used to locate them. In addition, chromosome arms without crossovers are listed with the 5' and 3' SNPs available for that chromosome arm.

Fly	Chr	Event	Crossover	5’ SNP	3’ SNP	5’ Genotype	3’ Genotype
**NDJ_01**	chr2L	MII	single	1817675	1817854	het	w1118
**NDJ_02**	chr2L	MII	none	5390	23455589	w1118	w1118
**NDJ_03**	chr2L	MI	single	2684283	2716561	w1118	het
**NDJ_04**	chr2L	MII	none	5390	23504712	OregonR	OregonR
**NDJ_05**	chr2L	MII	double	5396210	5396718	OregonR	het
**NDJ_05**	chr2L	MII	double	14095609	14100089	het	OregonR
**NDJ_06**	chr2L	MI	single	11232065	11298978	OregonR	het
**NDJ_07**	chr2L	MI	single	10508774	10513393	OregonR	het
**NDJ_08**	chr2L	MII	single	2992061	2999328	het	OregonR
**NDJ_09**	chr2L	MI	none	5904	23396898	het	het
**NDJ_10**	chr2L	MI	none	5390	23459270	het	het
**NDJ_11**	chr2L	MI	none	5390	23398207	het	het
**NDJ_12**	chr2L	MII	none	5390	23506600	OregonR	OregonR
**NDJ_13**	chr2L	MI	none	5390	23506583	het	het
**NDJ_14**	chr2L	MI	none	5390	23506605	het	het
**NDJ_15**	chr2L	MI	none	5390	23502997	het	het
**NDJ_17**	chr2L	MII	single	13844046	13852353	het	w1118
**NDJ_19**	chr2L	MI	none	106766	23398207	het	het
**NDJ_20**	chr2L	MI	none	5390	23506605	het	het
**NDJ_21**	chr2L	MI	none	12275	23504712	het	het
**NDJ_25**	chr2L	MI	none	8263	23506605	het	het
**NDJ_27**	chr2L	MI	single	10123127	10135970	w1118	het
**NDJ_29**	chr2L	MI	none	7556	23475821	het	het
**NDJ_31**	chr2L	MII	single	6299992	6303251	het	OregonR
**NDJ_35**	chr2L	MII	single	7898787	7947413	het	OregonR
**NDJ_37**	chr2L	MI	none	31003	23494115	het	het
**NDJ_38**	chr2L	MI	none	5598	23396898	het	het
**NDJ_39**	chr2L	MI	none	5390	23396898	het	het
**NDJ_40**	chr2L	MI	none	5390	23398207	het	het
**NDJ_41**	chr2L	MI	single	7069171	7069479	w1118	het
**NDJ_42**	chr2L	MI	double	7154399	7154554	het	w1118
**NDJ_42**	chr2L	MI	double	9646052	9650605	w1118	het
**NDJ_44**	chr2L	MI	none	7542	23398207	het	het
**NDJ_45**	chr2L	MI	none	5390	23398207	het	het
**NDJ_46**	chr2L	MI	none	5390	23396898	het	het
**NDJ_47**	chr2L	MI	none	5904	23398207	het	het
**NDJ_48**	chr2L	MI	single	2312135	2312580	OregonR	het
**NDJ_49**	chr2L	MI	none	5390	23494115	het	het
**NDJ_50**	chr2L	MI	none	7542	23398207	het	het
**NDJ_51**	chr2L	MI	none	5390	23398207	het	het
**NDJ_52**	chr2L	MII	single	14032703	14039830	het	w1118
**NDJ_53**	chr2L	MI	single	15619487	15629789	w1118	het
**NDJ_54**	chr2L	MI	single	3860389	3861629	w1118	het
**NDJ_55**	chr2L	MII	none	8263	23396898	OregonR	OregonR
**NDJ_56**	chr2L	MI	none	5465	23434569	het	het
**NDJ_57**	chr2L	MI	none	5390	23475821	het	het

**NDJ_58**	chr2L	MII	single	5533553	5534331	het	OregonR
**NDJ_59**	chr2L	MII	single	7118335	7120101	het	OregonR
**NDJ_60**	chr2L	MI	none	5390	23493402	het	het
**NDJ_61**	chr2L	MI	none	5390	23398207	het	het
**NDJ_62**	chr2L	MI	none	5390	23398207	het	het
**NDJ_63**	chr2L	MI	none	5390	23499203	het	het
**NDJ_64**	chr2L	MI	single	10606141	10606234	OregonR	het
**NDJ_65**	chr2L	MII	single	13873657	13875340	het	w1118
**NDJ_66**	chr2L	MI	none	7902	23506605	het	het
**NDJ_67**	chr2L	MI	none	5390	23506605	het	het
**NDJ_68**	chr2L	MI	none	5390	23506605	het	het
**NDJ_69**	chr2L	MI	single	3816205	3817502	w1118	het
**NDJ_70**	chr2L	MI	none	5390	23506605	het	het
**NDJ_71**	chr2L	MI	none	5390	23506605	het	het
**NDJ_72**	chr2L	MI	single	10147030	10147336	OregonR	het
**NDJ_73**	chr2L	MI	single	10045678	10046050	het	w1118
**NDJ_75**	chr2L	MI	none	5390	23506605	het	het
**NDJ_76**	chr2L	MI	none	5390	23427141	het	het
**NDJ_77**	chr2L	MI	none	5390	23502997	het	het
**NDJ_01**	chr2R	MII	single	16246587	16247087	w1118	both
**NDJ_02**	chr2R	MII	none	15841	25286255	w1118	w1118
**NDJ_03**	chr2R	MI	single	16356619	16357259	both	OregonR
**NDJ_04**	chr2R	MII	single	12995982	12998307	OregonR	w1118
**NDJ_05**	chr2R	MII	single	5848549	5849503	OregonR	het
**NDJ_06**	chr2R	MI	none	21087	25285566	het	het
**NDJ_07**	chr2R	MI	none	20833	25286160	het	het
**NDJ_08**	chr2R	MII	single	15667971	15670144	OregonR	w1118
**NDJ_09**	chr2R	MI	none	79674	25286255	het	het
**NDJ_10**	chr2R	MI	single	8582689	8583457	het	OregonR
**NDJ_11**	chr2R	MI	none	15841	25286255	het	het
**NDJ_12**	chr2R	MII	single	23266277	23267091	OregonR	het
**NDJ_13**	chr2R	MI	none	15841	25286160	het	het
**NDJ_14**	chr2R	MI	double	11636529	11643228	het	w1118
**NDJ_14**	chr2R	MI	double	15857846	15859435	w1118	het
**NDJ_15**	chr2R	MI	single	14734209	14736682	het	w1118
**NDJ_17**	chr2R	MII	none	16042	25286160	w1118	w1118
**NDJ_19**	chr2R	MI	single	16959418	17021394	het	w1118
**NDJ_20**	chr2R	MI	single	4141119	4141675	het	OregonR
**NDJ_21**	chr2R	MI	none	16004	25286160	het	het
**NDJ_25**	chr2R	MI	single	11682791	11682985	het	w1118
**NDJ_27**	chr2R	MI	single	6632226	6655417	het	w1118
**NDJ_29**	chr2R	MI	single	22056218	22136086	het	w1118
**NDJ_31**	chr2R	MII	single	12416521	12427700	OregonR	het
**NDJ_35**	chr2R	MII	none	33347	25286160	OregonR	OregonR
**NDJ_37**	chr2R	MI	none	19454	25286160	het	het
**NDJ_38**	chr2R	MI	none	32438	25285408	het	het
**NDJ_39**	chr2R	MI	none	35957	25286160	het	het
**NDJ_40**	chr2R	MI	single	10206737	10207903	het	w1118
**NDJ_41**	chr2R	MI	none	32438	25285566	het	het
**NDJ_42**	chr2R	MI	none	15841	25283162	het	het
**NDJ_44**	chr2R	MI	none	15841	25286160	het	het
**NDJ_45**	chr2R	MI	double	10000005	10000425	het	OregonR

**NDJ_45**	chr2R	MI	double	21295996	21303672	OregonR	het
**NDJ_46**	chr2R	MI	none	20833	25286255	het	het
**NDJ_47**	chr2R	MI	none	16042	25286071	het	het
**NDJ_48**	chr2R	MI	none	16004	25286255	het	het
**NDJ_49**	chr2R	MI	none	15841	25286255	het	het
**NDJ_50**	chr2R	MI	single	15567912	15568257	het	w1118
**NDJ_51**	chr2R	MI	none	16004	25286255	het	het
**NDJ_52**	chr2R	MII	none	15841	25286160	w1118	w1118
**NDJ_53**	chr2R	MI	none	32438	25286071	het	het
**NDJ_54**	chr2R	MI	none	15841	25285566	het	het
**NDJ_55**	chr2R	MII	none	15841	25286255	OregonR	OregonR
**NDJ_56**	chr2R	MI	none	20833	25286255	het	het
**NDJ_57**	chr2R	MI	single	6512541	6513791	het	OregonR
**NDJ_58**	chr2R	MII	single	20983414	20984314	OregonR	het
**NDJ_59**	chr2R	MII	single	18805462	11808076	OregonR	het
**NDJ_60**	chr2R	MI	none	16004	25286255	het	het
**NDJ_61**	chr2R	MI	none	19454	25286255	het	het
**NDJ_62**	chr2R	MI	none	15841	25286255	het	het
**NDJ_63**	chr2R	MI	none	15841	25286255	het	het
**NDJ_64**	chr2R	MI	single	12594607	12596624	het	OregonR
**NDJ_65**	chr2R	MII	none	15841	25286160	w1118	w1118
**NDJ_66**	chr2R	MI	none	19454	25286071	het	het
**NDJ_67**	chr2R	MI	single	16973112	16973532	het	w1118
**NDJ_68**	chr2R	MI	single	6512541	6513791	het	OregonR
**NDJ_69**	chr2R	MI	none	15841	25286255	het	het
**NDJ_70**	chr2R	MI	none	61774	25286255	het	het
**NDJ_71**	chr2R	MI	single	11655359	11658304	het	w1118
**NDJ_72**	chr2R	MI	single	5885264	5888987	het	het
**NDJ_73**	chr2R	MI	single	6655417	6655600	het	w1118
**NDJ_75**	chr2R	MI	none	15841	25286160	het	het
**NDJ_76**	chr2R	MI	single	17657948	17680813	het	w1118
**NDJ_77**	chr2R	MI	single	21226452	21231310	het	OregonR
**NDJ_01**	chr3L	MII	single	11636486	11637757	OregonR	w1118
**NDJ_02**	chr3L	MII	none	94773	28098081	OregonR	OregonR
**NDJ_03**	chr3L	MI	none	96034	28096994	w1118	w1118
**NDJ_04**	chr3L	MII	none	92693	28059767	OregonR	OregonR
**NDJ_05**	chr3L	MII	none	92693	28098081	OregonR	OregonR
**NDJ_08**	chr3L	MII	single	5658632	5659192	w1118	OregonR
**NDJ_11**	chr3L	MI	single	11732379	11732322	w1118	OregonR
**NDJ_12**	chr3L	MII	none	96034	28059767	w1118	w1118
**NDJ_13**	chr3L	MI	none	96034	28098081	OregonR	OregonR
**NDJ_14**	chr3L	MI	none	96034	28098081	OregonR	OregonR
**NDJ_15**	chr3L	MI	single	2725704	2727348	OregonR	w1118
**NDJ_17**	chr3L	MII	single	18472050	18473642	OregonR	w1118
**NDJ_19**	chr3L	MI	none	108486	28059767	OregonR	OregonR
**NDJ_20**	chr3L	MI	single	1674558	1697025	OregonR	w1118
**NDJ_21**	chr3L	MI	none	108486	28086478	OregonR	OregonR
**NDJ_25**	chr3L	MI	none	123416	28096994	OregonR	OregonR
**NDJ_27**	chr3L	MI	single	11398421	11427440	w1118	OregonR
**NDJ_29**	chr3L	MI	none	96034	28079976	OregonR	OregonR
**NDJ_31**	chr3L	MII	single	8559550	8563673	w1118	OregonR
**NDJ_35**	chr3L	MII	none	123416	28059767	OregonR	OregonR

**NDJ_37**	chr3L	MI	none	97623	28096994	w1118	w1118
**NDJ_45**	chr3L	MI	single	10936541	10946901	w1118	OregonR
**NDJ_49**	chr3L	MI	none	96034	28097345	OregonR	OregonR
**NDJ_56**	chr3L	MI	none	94773	28098081	OregonR	OregonR
**NDJ_57**	chr3L	MI	single	5435955	5439025	w1118	OregonR
**NDJ_58**	chr3L	MII	single	8386547	8405872	w1118	OregonR
**NDJ_59**	chr3L	MII	none	94773	28011895	OregonR	OregonR
**NDJ_60**	chr3L	MI	none	96034	28013143	w1118	w1118
**NDJ_62**	chr3L	MI	single	1351983	1354456	w1118	OregonR
**NDJ_63**	chr3L	MI	none	96034	28059767	w1118	w1118
**NDJ_69**	chr3L	MI	none	143083	28096994	w1118	w1118
**NDJ_01**	chr3R	MII	single	23042806	23043025	w1118	OregonR
**NDJ_02**	chr3R	MII	none	70385	32068674	OregonR	OregonR
**NDJ_03**	chr3R	MI	single	29459771	29460328	w1118	OregonR
**NDJ_04**	chr3R	MII	none	70385	32068674	OregonR	OregonR
**NDJ_05**	chr3R	MII	none	2377	32068674	OregonR	OregonR
**NDJ_08**	chr3R	MII	single	17666451	17668166	OregonR	OregonR
**NDJ_11**	chr3R	MI	none	109382	32068510	OregonR	OregonR
**NDJ_12**	chr3R	MII	none	87440	32022965	w1118	w1118
**NDJ_13**	chr3R	MI	none	2377	32056534	OregonR	OregonR
**NDJ_14**	chr3R	MI	none	70385	32022965	OregonR	OregonR
**NDJ_15**	chr3R	MI	none	88849	32016427	w1118	w1118
**NDJ_17**	chr3R	MII	single	23083408	23141711	w1118	OregonR
**NDJ_19**	chr3R	MI	none	22990	32078824	OregonR	OregonR
**NDJ_20**	chr3R	MI	none	35917	32021038	w1118	w1118
**NDJ_21**	chr3R	MI	none	22990	32022965	OregonR	OregonR
**NDJ_25**	chr3R	MI	none	2377	32021038	OregonR	OregonR
**NDJ_27**	chr3R	MI	none	22990	32025863	OregonR	OregonR
**NDJ_29**	chr3R	MI	none	59018	32078824	OregonR	OregonR
**NDJ_31**	chr3R	MII	none	40317	32066263	OregonR	OregonR
**NDJ_35**	chr3R	MII	none	40317	31920756	OregonR	OregonR
**NDJ_37**	chr3R	MI	none	40317	31995116	w1118	w1118
**NDJ_45**	chr3R	MI	none	174988	32066263	OregonR	OregonR
**NDJ_49**	chr3R	MI	single	22591407	22595119	OregonR	w1118
**NDJ_56**	chr3R	MI	none	106481	32058725	OregonR	OregonR
**NDJ_57**	chr3R	MI	single	22831832	22832450	OregonR	w1118
**NDJ_58**	chr3R	MII	none	174988	32058725	OregonR	OregonR
**NDJ_59**	chr3R	MII	none	108395	31986239	OregonR	OregonR
**NDJ_60**	chr3R	MI	single	30619537	30766179	w1118	OregonR
**NDJ_61**	chr3R	MI	none	108395	32063517	w1118	w1118
**NDJ_61**	chr3R	MI	single	24011807	24014458	w1118	OregonR
**NDJ_62**	chr3R	MI	none	106481	32078824	OregonR	OregonR
**NDJ_63**	chr3R	MI	none	70604	32021038	w1118	w1118
**NDJ_69**	chr3R	MI	none	70604	31924804	w1118	w1118
**NDJ_01**	chrX	MII	single	3555500	3558647	OregonR	w1118
**NDJ_02**	chrX	MII	single	4350321	4361286	w1118	OregonR
**NDJ_03**	chrX	MI	double	5680185	5775463	OregonR	w1118
**NDJ_03**	chrX	MI	double	13303838	13321915	w1118	OregonR
**NDJ_04**	chrX	MII	single	7271843	7894153	w1118	OregonR
**NDJ_05**	chrX	MII	single	8913250	8928098	w1118	OregonR
**NDJ_08**	chrX	MII	none	57879	23532498	OregonR	OregonR
**NDJ_11**	chrX	MI	single	4325635	4629119	w1118	OregonR

**NDJ_12**	chrX	MII	single	5702847	5841699	OregonR	w1118
**NDJ_13**	chrX	MI	single	7109577	7135728	w1118	OregonR
**NDJ_14**	chrX	MI	single	14138052	14171936	w1118	OregonR
**NDJ_15**	chrX	MI	none	57879	23532498	w1118	w1118
**NDJ_20**	chrX	MI	none	61933	23532498	OregonR	OregonR
**NDJ_21**	chrX	MI	none	57879	23532498	w1118	w1118
**NDJ_27**	chrX	MI	single	12047003	12620586	OregonR	w1118
**NDJ_29**	chrX	MI	none	56267	23532498	OregonR	OregonR
**NDJ_49**	chrX	MI	single	2751983	2769277	w1118	OregonR
**NDJ_56**	chrX	MI	single	19367568	19376820	w1118	OregonR
**NDJ_57**	chrX	MI	single	11618071	11928529	w1118	OregonR
**NDJ_58**	chrX	MII	double	5867507	6285251	w1118	OregonR
**NDJ_58**	chrX	MII	double	14470033	14568250	OregonR	w1118
**NDJ_60**	chrX	MI	none	63364	23532498	w1118	w1118
**NDJ_61**	chrX	MI	single	7251508	7894153	w1118	OregonR
**NDJ_62**	chrX	MI	none	57879	23532498	OregonR	OregonR
**NDJ_63**	chrX	MI	single	3298174	3379994	w1118	OregonR
